# Comments on Meo *et al*. Association of Exposure to Radio-Frequency Electromagnetic Field Radiation (RF-EMFR) Generated by Mobile Phone Base Stations with Glycated Hemoglobin (HbA1c) and Risk of Type 2 Diabetes Mellitus. *Int. J. Environ. Res. Public Health*, 2015, *12*, 14519–14528

**DOI:** 10.3390/ijerph13030261

**Published:** 2016-02-26

**Authors:** Seyed Alireza Mortazavi, Ghazal Mortazavi, Seyed Mohammad Javad Mortazavi

**Affiliations:** 1 Student Research Committee, School of Medicine, ShirazUniversity of Medical Sciences, Shiraz 7134845794, Iran; a.mortazavi.72@gmail.com; 2 Tangestan Health Network, Bushehr University of Medical Sciences, Bushehr 7551866897, Iran; qaz.mortazavi@gmail.com; 3 Ionizing and Non-Ionizing Radiation Protection Research Center (INIRPRC), ShirazUniversity of Medical Sciences, Shiraz 7134845794, Iran; 4 Medical Physics and Medical Engineering Department, School of Medicine, ShirazUniversity of Medical Sciences, Shiraz 7134845794, Iran

With great interest and enthusiasm, we have read the article by Meo *et al*. entitled “Association of Exposure to Radio-Frequency Electromagnetic Field Radiation (RF-EMFR) Generated by Mobile Phone Base Stations with Glycated Hemoglobin (HbA1c) and Risk of Type 2 Diabetes Mellitus” that is published in the latest issue of the International Journal of Environmental Research and Public Health [1]. These researchers investigated in this article the effect of exposure to radiofrequency electromagnetic fields (RF-EMFs) emitted by mobile phone base stations (MPBSs) on the glycated hemoglobin (HbA1c) and the risk of type 2 diabetes mellitus. They found significant differences between the mean levels of HbA1c in the students who were exposed to high RF-EMFs compared to those of the students who were exposed to low RF-EMFs. Furthermore, they reported that the students who were exposed to high RF-EMFs had a significantly increased risk of type 2 diabetes mellitus compared to the students who were exposed to low RF-EMFs.

Over the past several years, our laboratories at INIRPRC have expanded their focus on studying the health effects of exposure to some common and/or occupational sources of electromagnetic fields such as cellular phones, mobile base stations, mobile phone jammers, laptop computers, radars, dentistry cavitrons and MRI. It is worth noting that we have addressed both detrimental and beneficial/stimulatory effects of exposure to electromagnetic fields (these data are available in our published review articles [2,3]). Although the topic of the well-structured paper authored by the authors is challenging, this paper has some shortcomings. First of all, these researchers stated that they recruited a total of 159 students (96 male students from school-1 with the RF-EMFs level of 9.601 nW/cm^2^, and 63 male students from school-2 with the RF-EMFs level of 1.909 nW/cm^2^. As the difference between these RF-EMFs levels is less than one order of magnitude, the findings may be simply affected by various confounding factors. It is worth mentioning that the variations of RF-EMF level in our daily life are much greater. The second shortcoming of this paper comes from ignoring the students’ exposure to different EMF sources at their homes. Nowadays, students are among the frequent users of mobile phones. A recent study showed that children and teenagers who need to communicate nearly 24 h a day, are the largest group of smart phone users. This study claims that nowadays cell phones and tablets may be seen in the hands of children as little as two years in age [4]. They frequently use their mobile phones or tablets for surfing the web, checking social networks and playing games than making phone calls. On the other hand, Wireless Fidelity (Wi-Fi) routers are widely used in all houses (residents of small apartments are usually exposed to radiations emitted by several Wi-Fi routers). The rapidly increasing exposure to the Wi-Fi wireless radiation has raised great public concerns especially for young people [5]. Therefore, the differences observed in the study of Meo *et al*. may be due to the different “at home” RF-EMF exposure pattern in these 2 groups of students. It is worth noting that socio-economic factors can intensify these “at home’ exposures. We hope these comments help researchers better evaluate the challenging issue of the health effects of exposure to RF-EMFs emitted by rapidly increasing cell phone base stations.
